# Computer-Assisted Interpretation of the EEG Background Pattern: A Clinical Evaluation

**DOI:** 10.1371/journal.pone.0085966

**Published:** 2014-01-24

**Authors:** Shaun S. Lodder, Jessica Askamp, Michel J. A. M. van Putten

**Affiliations:** 1 Clinical Neurophysiology, MIRA-Institute for Biomedical Technology and Technical Medicine, University of Twente, The Netherlands; 2 Department of Neurology and Clinical Neurophysiology, Medisch Spectrum Twente, Enschede, The Netherlands; University of Maryland, College Park, United States of America

## Abstract

**Objective:**

Interpretation of the EEG background pattern in routine recordings is an important part of clinical reviews. We evaluated the feasibility of an automated analysis system to assist reviewers with evaluation of the general properties in the EEG background pattern.

**Methods:**

Quantitative EEG methods were used to describe the following five background properties: posterior dominant rhythm frequency and reactivity, anterior-posterior gradients, presence of diffuse slow-wave activity and asymmetry. Software running the quantitative methods were given to ten experienced electroencephalographers together with 45 routine EEG recordings and computer-generated reports. Participants were asked to review the EEGs by visual analysis first, and afterwards to compare their findings with the generated reports and correct mistakes made by the system. Corrected reports were returned for comparison.

**Results:**

Using a gold-standard derived from the consensus of reviewers, inter-rater agreement was calculated for all reviewers and for automated interpretation. Automated interpretation together with most participants showed high (kappa > 0.6) agreement with the gold standard. In some cases, automated analysis showed higher agreement with the gold standard than participants. When asked in a questionnaire after the study, all participants considered computer-assisted interpretation to be useful for every day use in routine reviews.

**Conclusions:**

Automated interpretation methods proved to be accurate and were considered to be useful by all participants.

**Significance:**

Computer-assisted interpretation of the EEG background pattern can bring consistency to reviewing and improve efficiency and inter-rater agreement.

## Introduction

Scalp EEG is used in a wide range of clinical settings to obtain a non-invasive measurement of cortical brain activity. Having a higher temporal resolution and being more affordable, portable and widely available than fMRI and MEG, its uses range from diagnostics and monitoring for outpatient recordings, to continuous monitoring in the ICU. The recordings are typically analyzed by visual inspection of the signals in their raw form, and apart from being a time consuming and error prone task which may lead to missed events, this can also result in high inter- and intra-rater variability depending on the level of experience and degree of concentration of the reviewer [1].

An important part of EEG reviews is the analysis of the background pattern. For routine outpatient recordings this plays an important part in epilepsy diagnostics [2], [3], clinical psychiatry [4] and the diagnosis of neurodegenerative diseases [5]–[7]. Also, continuous monitoring of the background pattern in the ICU can alert medical staff of sudden changes that require immediate intervention [8]–[10], thereby changing its role from passive to an active tool for improving the outcomes of the critically ill.

Quantitative EEG analysis (QEEG) and structured reports have been proposed to lessen the burden of visual reviews and to add more consistency during reporting [3], [11]–[14]. Standard guidelines for writing EEG reports state that objective observations of the EEG properties should be made first, followed by the conclusions drawn from the reviewer based on these observations (see Guideline 7 for writing EEG reports provided by the American Clinical Neurophysiology Society). As such, quantitative analysis is well suited during the first phase, i.e. by assisting in the objective description of all background properties in a consistent manner. Given that other factors such as medication and patient history are not known or taken into consideration by quantitative analysis, conclusions drawn after initial observations should be left to the reviewer.

As shown in [15], inter-rater agreement for describing EEG observations can be improved if reviewers agree to follow a clear set of guidelines in reporting their findings. These guidelines should be obtained from the general consensus of experienced electroencephalographers themselves. In recent work reported in [14], the authors show on how such a set of guidelines and definitions are being constructed as part of a pan-European project with the goal of providing more consistency and structure for the reporting in clinical EEG reviews [11], [14], [16].

Many types of quantitative EEG features have been proposed to describe specific properties in the EEG. These include statistical measures such as variance, kurtosis and skewness [17], [18], non-linear energy operators [19], small-world networks and functional connectivity [20], [21], synchrony [22], [23], entropy [24], [25], power ratios [9], [26], bi-spectral index [27], and left-right symmetry [28]. Despite the variety of complex features available, relatively simple measures can be used to describe many of the background properties of an EEG. Example features are the presence or absence of certain rhythmic components, power ratios between delta-, theta-, alpha- and beta-bands, and the power distribution over the scalp. The importance of each background property will vary based on the reason for recording, but in general, a description of the background pattern is of significant importance for any review.

In a previous study, we described an automated system based on quantitative features that can be used to assist with the interpretation of the EEG background pattern [29]. Based on the methods described in [12], this system uses quantitative analysis to estimate properties for the posterior dominant rhythm, reactivity, anterior-posterior gradients, presence of diffuse slow wave activity, and symmetry, and then determines if these properties are abnormal or fall within the normal range. Together with constructing an automated report based on the outcome of these features, the system also shows the quantitative properties to the user in a simple and intuitive manner.

The results in [29] showed that quantitative features can be used to find accurate measures of the common EEG background properties. It was however also shown that regardless of good predictions, the system was not reliable enough to match all outcomes from the diagnostic reports based on visual analysis. It can therefore only serve to assist during a review and not replace visual analysis. A reason for lower agreement with the diagnostic reports could also have been related to a lack of consistent reporting from visual analysis that lead to higher inter- and intra-rater variability, and a follow-up goal was therefore to measure inter-rater agreement in standard reports for these properties.

Although some background properties have more relevance than others depending on the clinical setting and reason for recording, all of these considered form part of the common properties described in most routine EEG reports. The goal of this study was to evaluate this system in routine outpatient EEG recordings by sending it to a group of qualified and experienced electroencephalographers, and to determine if there is any additional benefit in computer assisted interpretation together with conventional EEG reviews. Apart from evaluating the accuracy of the automated system, we also wanted to measure the inter-rater agreement between reviewers, and in addition to this obtain feedback from participants about their experience and perceived importance of using quantitative features for future reviews. Fast and accurate interpretation of the background pattern by means of computer assisted analysis can save time for reviewers, reduce the costs of analysis, bring consistency and completeness to EEG reports, reduce inter-rater variability, and allow for both experienced and less experienced electroencephalographers to benefit from additional visualizations of the EEG by presenting it in a simpler and more intuitive manner.

## Methods

### Subjects and Data

The dataset used for this study consisted of 45 anonymized routine scalp EEGs, each 20–30 min in length. Regarding ethical approval for using the EEG data in this study, according to Dutch law, researchers do not need to consult a medical ethical committee if patient data has been obtained as part of routine patient care. Furthermore, patient consent is not needed for additional use of these data for further scientific research if the data has been anonymized. These statements have been confirmed by our medical ethical committee. All recordings were obtained from the Medisch Spectrum Twente hospital in the Netherlands. Original diagnostic reports were used to find example EEGs in such a way that both normal and abnormal occurrences were available for each background property. Apart from ensuring that both normal and abnormal occurrences exist, the dataset was chosen randomly with recording dates ranging over 7 years. Our selection was unbiased towards the number of artifacts each recording contained, and subject ages ranged from 10 to 88 years (mean 53.2). Patients were awake during the recording and a standard 20–30 min protocol was used, which included hyperventilation and photic stimulation. None of the recordings were sleep-deprived EEGs. The EEGs were recorded at a sample rate of either 250 or 256 Hz with the Brainlab EEG system, and Ag-AgCl electrode caps were used with electrodes placed according to the 10–20 system. Impedances were kept below 5 kΩ to reduce polarization effects.

### Automated Interpretation of the Background Pattern

As a first step, quantitative features were calculated for each EEG in the dataset. A brief outline on the calculation of each quantitative feature is provided in Appendix S1, and a detailed description can be found in [29]. Five background properties were considered, and based on the threshold values provided in Appendix S1, an automated description of each property was obtained. The five background properties were: *i*) the posterior dominant rhythm frequency and *ii*) its reactivity, *iii*) anterior-posterior gradients, *iv*) asymmetries, and *v*) the presence or absence of diffuse slow-wave activity. In the case of asymmetry, the system also determined the affected regions in which asymmetries appeared. Available options for this were the left and right frontal, central, temporal, parietal, and occipital regions. The calculated findings were stored in diagnostic reports that were later presented to the reviewers for verification. To make the outcomes compatible with visual reviews and easier to compare, set categories were defined in the reports for the outcomes of each property. These categories are shown in Table 1.

**Table 1:** To make outcomes comparable with visual reviews, set categories were defined in the reports for the outcomes of each property.

*Posterior dominant rhythm (PDR)*		
None	Normal	Abnormal
*PDR frequency*		
*f*(Hz)		
*Reactivity*		
Substantial	Moderate	Low or absent
*AP-Gradient*		
Normal	Moderate	Abnormal or deviant
*Abnormal diffuse slow waves*		
Yes present	None	
*Asymmetries*		
None	One or more	
*Affected asymmetry region*		
Left	Right	None

### Visual Inspection and Confirmation

**Figure 1:**
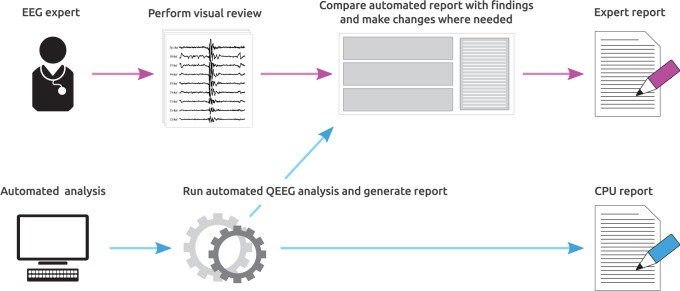
Outline of the study. For each of the 45 recordings, participants were asked to open the EEG in the conventional way (Fig. 2) and review five background properties by visual inspection. After this, they were asked to open a new window showing a summary of the quantitative features (Fig. 3), and to correct the mistakes made in a report generated by automated interpretation.

**Figure 2:**
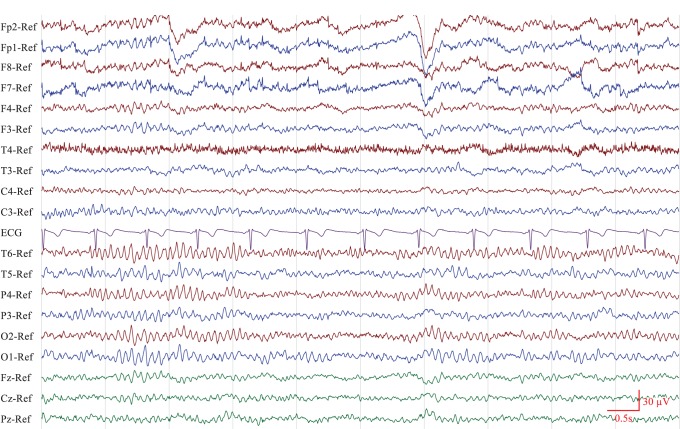
Before evaluating the automated reports and correcting mistakes made by quantitative analysis, participants were asked to review the EEG conventionally by visual inspection of the recording in its raw form.

**Figure 3:**
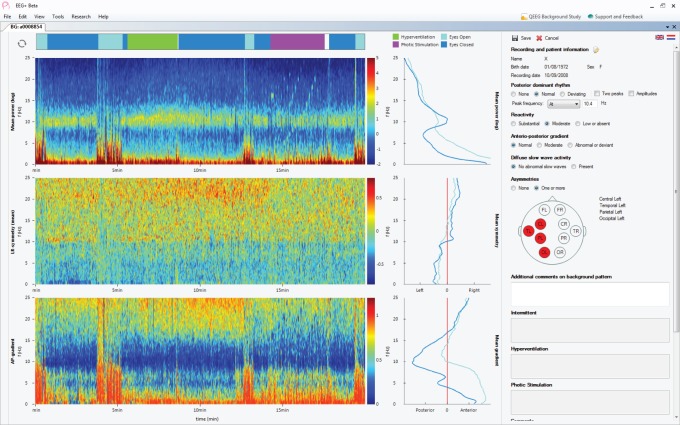
After visually reviewing the EEG in its raw form, participants were shown a quantitative EEG display which summarizes the entire recording into a single window. Using the report provided in this window (right), participants were asked to correct the automated interpretation where needed and afterwards upload the corrected reports for comparison.

To compare automated reporting with visual analysis, the same dataset was sent to ten certified and experienced electroencephalographers across multiple centers within the Netherlands, together with a set of instructions and examples on how to interpret the displayed quantitative features. Fig. 1 shows an outline of the protocol followed by each participant. For each of the 45 recordings, participants were asked to first open the EEG as usual (i.e. ten second pages of raw time series data) as shown in Fig. 2, scroll through the recording, and review the five background properties by visual inspection. After this, they were asked to open a new window in the software application showing the same recording, but this time using quantitative features instead of the raw time series data. In this new window was also the automatically generated report of the EEG background properties. An example of this display is shown in Fig. 3. The participants were then asked to verify the outcome of the automatically generated reports with their interpretation from visual inspection and make changes to it where automated analysis was wrong. To edit the reports, a user interface with predefined checkboxes for the outcome of each property was provided, as shown on the right of Fig 3. Possible outcomes for each property are summarized in Table 1. Together with this, participants also had the opportunity to compare their findings from visual analysis with the quantitative features displayed on the left side of the quantitative analysis screen (Fig. 3). After updating the reports and confirming that all findings were correct, the reports were uploaded to our server and later analyzed for comparison.

### User Experience

Given that the system would also require general acceptance by reviewers to become clinically relevant, an attempt was made to measure the user experience and willingness of participants to include assisted interpretation into their routine reviewing procedure. To obtain a quantitative measure for this, participants were asked five questions after evaluating all the EEGs. These questions, together with the respondent averages, are shown in Table 2. The questions were focused on obtaining information about the reviewers’ previous experience with quantitative EEG analysis, its ease of use, and their perceived importance of quantitative EEG analysis in future reviews.

**Table 2:** Five questions were asked to each participant after all EEGs were reviewed.

*1. Before this study, how often have you used any form of quantitative analysis methods to help you with*					
*(a) Routine EEG reviews?*					
	Never (20%)	Rarely (0%)	Occasionally (40%)	Often (20%)	Always (20%)
*(b) Long-term EEG reviews?*					
	Never (20%)	Rarely (10%)	Occasionally (30%)	Often (30%)	Always (10%)
*2. How often did the quantitative analysis figures or generated reports alter your conclusions based on visual interpretation of the EEG (eg. picked up a missed asymmetry or different degree of reactivity)?*					
	Never (0%)	Rarely (20%)	Occasionally (80%)	Often (0%)	Always (0%)
*3. Did you find the automatically generated reports useful during your review?*					
	Not at all (0%)	Rarely (0%)	Occasionally (50%)	Often (30%)	Most of the time (20%)
*4. In your opinion, do you think that compared to the standard way of reviewing EEGs, the added quantitative features will make you spend more time, less time, or the same amount of time to perform a review?*					
	More (0%)	Same (50%)	Less (50%)		
*5. Will you find it useful to have quantitative analysis methods assist you in every day interpretation of:*					
*(a) Routine EEGs*					
	No (0%)	Yes (100%)			
*(b) Long-term EEGs*					
	No (0%)	Yes (100%)			

## Results

To determine the feasibility of computer-assisted reviewing, the first important step was to determine the accuracy and robustness of the quantitative analysis methods. This was done by calculating the inter-rater agreement between the computer-generated reports and a gold standard, which was obtained by taking the most agreed upon outcome for each property in each of the recordings as given by the ten participants. Using the gold standard for comparison, the inter-rater agreement was calculated for both the automatically generated reports and for individual participants that served as a benchmark. Inter-rater agreements were calculated using Fleiss’ Kappa measure, and the results of these comparisons are shown in Table 3. Although no universally excepted rule exists for interpreting Kappa values, a popular guideline is provided by [30] and will be used here accordingly (see Table 4 for reference). The first ten rows in Table 3 show the inter-rater agreement between each participant and the gold standard for each of the background properties. Compared with the gold standard, nearly all participants showed substantial (0.61−0.80) and almost perfect (>0.81) agreement for all properties apart from the presence and location of asymmetries. For the presence of asymmetries, four participants showed moderate agreement (0.41−0.60) and the remaining six substantial agreement or higher. In reporting the location of the asymmetries, two participants showed moderate agreement and the remaining participants all substantial or almost perfect agreement. One participant did not specify the asymmetry regions. The last row of Table 3 shows the inter-rater agreement between the the gold standard and the automatically generated reports as obtained by automated analysis. Here we see that for the PDR, reactivity, anterior-posterior gradient and slowing, the system obtained almost perfect agreement with the gold standard. For asymmetries and asymmetry regions, the system obtained almost perfect and substantial agreement respectively. Also seen is that the system showed higher agreement with the gold standard than some reviewers.

**Table 3:** Inter-rater agreement between the gold standard and reviewers (R{i}), and the gold standard and automated analysis (CPU) respectively.

Reviewer	PDR	Reactivity	AP-Gradient	Slowing	Asymmetries	Asymmetry regions
R1	0.87	0.95	0.85	1.00	0.70	0.77
R2	0.87	0.61	0.58	0.81	0.54	0.71
R3	0.93	0.95	0.95	1.00	0.83	–
R4	0.63	0.68	0.69	0.80	0.76	0.86
R5	0.67	0.75	0.86	0.74	0.64	0.74
R6	0.87	0.64	0.63	0.85	0.44	0.59
R7	0.77	0.33	0.65	0.78	0.44	0.61
R8	0.93	0.91	0.86	1.00	0.55	0.50
R9	0.94	0.95	0.90	1.00	0.91	0.54
R10	0.88	0.56	0.82	1.00	0.66	0.62
CPU	0.93	0.95	0.95	1.00	0.83	0.77

The gold standard was based on the most agreed upon outcome for each property using reports from the participants, and the inter-rater agreement was measured using Fleiss’ Kappa.

**Table 4:** Interpretation of Kappa values as suggested by [30].

*κ*	Interpretation
<0	Poor agreement
0.01−0.20	Slight agreement
0.21−0.40	Fair agreement
0.41−0.60	Moderate agreement
0.61−0.80	Substantial agreement
0.81−1.00	Almost perfect agreement

Considering the questionnaire that participants were asked to complete at the end of the study, two said that they have never used quantitative analysis before or during a routine or long-term review, whereas the others have all used quantitative measures mostly on an occasional, regular or routine basis. Quantitative analysis also appears to be used with same rate in long-term recordings as in routine EEGs. Regarding the question asking participants if they found the automatically generated reports useful during their review, two said only rarely and the remaining eight occasionally. Half of the participants also said that with the use of quantitative features they expect to spend less time to perform a review, whereas the other half said that they expected to spend the same amount of time as with a conventional review. None of the participants expected to spend more time. Lastly, asking the participants if they would find it useful to have quantitative analysis methods assist them during their daily interpretation of both routine and long-term EEGs, all of them responded with yes.

## Discussion

This study investigated the clinical relevance of computer assisted interpretation of the EEG background pattern. Our specific goal was to test the quantitative analysis methods described in [29] and to determine if they bring added value to conventional reviewing procedures. To do this, an EEG dataset consisting of 45 routine scalp EEGs was given to both the automated interpretation system described in [29] and to ten experienced electroencephalographers for review. Using a gold standard derived from the reviewer reports, the inter-rater agreement was calculated for each participant and for the automatically generated reports by themselves.

From Table 3 we see that there was high inter-rater agreement between all reviewers for all of the background properties described, and only once was the inter-rater agreement less than moderate for any of the properties (reactivity for reviewer R7). Although the quantitative algorithms followed a specific set of guidelines to determine the difference between normal and abnormal properties as described in [29], participants were not explicitly asked to do the same. The reason for this is that in some cases, these guidelines may be too simplistic in nature to make accurate interpretations. We therefore allowed the reviewers to make interpretations based on their own experience, and for future use we intend to combine the guidelines used in [29] with the proposed guidelines given by [14], and will encourage users to use this instead to ensure conformity and standardization of EEG reports. Following the same set of guidelines may also help to improve the inter-rater agreement, as demonstrated in [15].

An important observation in Table 3 is that the automatically generated reports had higher agreement with the gold standard than many of the reviewer reports. This is an interesting finding, but should be interpreted carefully given that a bias was added by asking participants to correct the generated reports instead of letting them fill out the entire report by themselves. As a partial compensation to avoid this bias, participants were not shown the pre-calculated reports until after they drew their own conclusions based on visual inspection of the entire recording, as described in the methods and shown in the experiment outline in Fig.1. Due to the inclusion of a possible bias however, we cannot conclude that automated analysis is more in agreement with the gold standard than some reviewers, although it does point to the fact that automated analysis is more consistent with interpretation than some reviewers. To make a fair comparison between visual analysis and automated interpretation, a similar study will have to be done where participants are asked to fill out reports without receiving input from quantitative analysis. Our aim however is not to replace the reviewer but instead to assist him, and it was therefore chosen to perform the study in this way in order to also receive feedback from participants based on their experience of using computer assisted interpretation.

This study made use of partially structured reports to describe the EEG background properties. Although provision was not made for all the background features, an additional text box was available for reviewers to add additional comments or to expand their descriptions on specific properties if they wished to do so. Together with other improvements, more categorized properties can be added to provide a fully structured report, as shown by for example [11], [31]. Structured reports make it easier for reviewers to follow general guidelines in describing EEG properties, and helps them to perform their review of each property in a consistent manner, without leaving anything out.

Although the five questions asked to the participants at the end of the study were only general in nature, our objective with this was to measure the overall acceptance of computer assisted methods together a conventional EEG review. Automated interpretation of the general properties associated with the EEG background pattern has been suggested before [32]–[34], but up to now is still not widely accepted for routine clinical use. Based on the received answers from the questionnaire however, we do see that many participants make use of quantitative EEG measures in some way or another. Other quantitative systems are typically aimed at describing one or two specific properties in the EEG, for example markers pointing to neurodegenerative diseases [5], [35], [36] or psychiatric disorders [4], and trends showing burst-suppression rates or seizures in long-term ICU monitoring [9], [10]. Regarding the automatically generated reports for describing the general background properties of the EEG, most participants considered it to be useful during their review.

One of the important goals of this study was to try and improve the overall inter-rater agreement in describing the EEG background pattern. When participants were asked if the generated reports altered their initial conclusions based on visual analysis alone, most of them indicated that it had done so occasionally. This shows that although quantitative analysis may also make mistakes, it becomes useful as an assistant during reviews and helps to improve reviewer consistency and intra-rater reliability. Therefore, given that none of the reviewers will expect to take longer to perform their reviews by adding computer-assisted analysis, this approach may benefit the final outcome of an EEG review without reducing the reviewer efficiency. Given that the recording and review of EEGs are also one of the highest costs involved with neurological visits apart from MRI and EMG [37], [38], any improvement in efficiency should lead to a significant reduction in overall healthcare costs. All participants also indicated that they would find it useful to have quantitative analysis methods assist them with every day interpretation of routine and long-term EEGs, showing a very positive sign for the general acceptance of computer assisted reviewing.

The system presented here is of course far from perfect and many improvements can still be made to provide a better and more efficient user experience. For some features, in particular the detection of asymmetries, more detailed and also more accurate interpretations from the automated system are needed to improve the inter-rater agreement. Also, the interpretation of additional properties such as mu and beta rhythms, lambda waves, and response to hyperventilation and photic stimulation for example, are needed to to provide a complete description of the EEG background pattern. Although there was no selection bias in the chosen dataset regarding artifacts, it can be assumed that the system will become less accurate if too many artifacts exist. However, given our sample size of 45 EEGs that also included the normal amount of artifacts expected from a routine recording, the system appears to be fairly robust. In the case where artifacts would severely affect the performance of the system, they will clearly be seen on the summarized review screen (left side) as shown in Fig. 3. The reviewer should then be advised not to trust the outcome of the automated review.

It is important to keep in mind that, as also stated in [13], quantitative features and automated systems should remain transparent where possible and care should be taken not to over-complicate algorithms and thereby lose the confidence of the reviewer. Regarding the visualization of the quantitative features as shown in Fig. 3, some participants have also commented on non-intuitive parts of the display, leading to them making less use of it. Further work is therefore needed to improve the quantitative displays and to find more intuitive methods to clearly show and summarize the EEG background properties.

In summary, a successful and accurate implementation of computer assisted interpretation of the EEG background pattern can assist reviewers in their daily routine of reviewing EEGs. Together with the structured reports obtained by this system, this will bring more consistency to reviewing and further improve the inter-rater agreement. Simple and intuitive ways of showing quantitative features can also summarize and present the entire recording on a single display and thereby bring added benefits to both experienced and inexperienced reviewers alike, and in addition help to reduce the reviewing time significantly.

## Supporting Information

Appendix S1 **Quantitative background features.**
(PDF)
