# Supplementary material for: Computer-Assisted Interpretation of the EEG Background Pattern: A Clinical Evaluation
Source: PLoS One. 2014 Jan 24;9(1):e85966. doi: 10.1371/journal.pone.0085966 (PMC3901663; doi:10.1371/journal.pone.0085966)
Supplement: Appendix S1 — Quantitative background features. (PDF) [file pone.0085966.s001.pdf]

Appendix S1

Quantitative background features

Five background properties were evaluated and interpreted with quantitative measures in this study. They were: *i*) the posterior dominant rhythm frequency and *ii*) its reactivity, *iii*) anterior-posterior gradients, *iv*) asymmetries, and *v*) the presence or absence of diffuse slow-wave activity. A brief outline of the various properties are provided below, and for more details, the reader is referred to [29].

For the posterior dominant rhythm, a peak frequency was calculated over the occipital region by dividing each EEG into segments of five seconds each and using a curve-fitting approach to find the dominant peaks in the eyes-closed segments. To determine the current state that the patient was in, eyes open, eyes closed, hyperventilation, and photic stimulation events were extracted from the recording annotations and processed accordingly. Afterwards, the peak estimates of all segments were clustered together to determine the dominant frequency components, and from this an estimate of the posterior dominant peak frequency was obtained. A full description of this technique is described in [29].

After obtaining the peak dominant frequency, the reactivity of the posterior dominant rhythm was calculated by measuring the mean occipital power in a 0.5 Hz frequency band around that frequency when the eyes are open P_{EO} and and when the eyes are closed P_{EC}:

$$Q_{REAC} = 1 - \frac{P_{EO}}{P_{EC}}. \tag{1}$$

The reactivity was classified as substantial when $Q_{REAC} > 0.5$, moderate for $0.1 < Q_{REAC} < 0.5$, and low or absent if $Q_{REAC} < 0.1$.

Anterior posterior gradients were measured by calculating the mean power over the anterior P_{ant} and posterior regions P_{pos} of the scalp, and finding a normalized value that quantifies the contribution of P_{ant} to the total power:

$$Q_{APG} = \frac{P_{ant}}{P_{ant} + P_{ant}}. \tag{2}$$

The alpha power gradient was considered within normal range if Q_{APG} was lower than 0.4, moderately differentiated between 0.4 and 0.6, and abnormal or deviant for values above 0.6.

In a similar manner, asymmetries were found by comparing power differences between matching left- and right electrode pairs:

Lastly, the presence of diffuse slow-wave activity was calculated by comparing the power in lower theta and delta bands (2-8 Hz) P_{low} to a wider spectral band ranging from 2-25 Hz P_{wide}:

$$Q_{SLOWING} = \frac{P_{low}}{P_{wide}}. \tag{3}$$

Diffuse slow-wave activity was considered to be present if $Q_{SLOWING} > 0.6$, i.e., less than 40% of the power was above 8 Hz.
